# General anesthesia with spontaneous breathing and laryngeal mask airway intubation combined with peripheral nerve block in orthopedic surgery: a retrospective study

**DOI:** 10.3389/fmed.2025.1584437

**Published:** 2025-09-24

**Authors:** Jiahui Tu, Piao Peng, Zhaodong Xiong

**Affiliations:** Department of Anesthesiology, The First People’s Hospital of Huzhou, First Affiliated Hospital of Huzhou University, Huzhou, China

**Keywords:** spontaneous breathing, laryngeal mask airway, peripheral nerve block, general anesthesia, postoperative pulmonary complications, orthopedic surgery

## Abstract

**Objectives:**

To investigate the effect of general anesthesia with spontaneous breathing laryngeal mask airway (LMA) intubation combined with a peripheral nerve block (PNB) in orthopedic surgery through a retrospective study.

**Methods:**

Data from 160 adult patients (American Society of Anesthesiologists grade 1–3) who underwent elective orthopedic surgery under general anesthesia with LMA intubation combined with PNB were retrospectively analyzed. All patients received ultrasound-guided PNB before general anesthesia. Among them, 78 patients were assigned to the spontaneous breathing group and 82 patients to the pressure-controlled ventilation group. The parameters compared included baseline characteristics, perioperative anesthetic drug dosages, anesthesia recovery time, adverse events during recovery, numerical rating scale score at 2 h postoperatively, postoperative pulmonary complications, hospitalization costs, and length of stay.

**Results:**

Both groups successfully completed the surgeries. Compared to the pressure-controlled ventilation group, the spontaneous breathing group exhibited significantly lower doses of fentanyl and rocuronium bromide (*p* < 0.001), shorter length of stay (*p* = 0.047), reduced incidence of postoperative hypertension in the post-anesthesia care unit (*p* < 0.001), and lower anesthesia costs (*p* < 0.001) and total hospitalization costs (*p* = 0.001). No statistically significant differences were observed between the two groups in baseline characteristics, anesthesia recovery time, incidence of postoperative hypoxemia or hypotension in the post-anesthesia care unit, numerical rating score at 2 h postoperatively, incidence of postoperative pulmonary complications, or surgical costs.

**Conclusion:**

General anesthesia with spontaneous breathing LMA intubation combined with PNB is safe and feasible for elective orthopedic surgeries in adults. This approach is beneficial in shortening the length of stay and reducing hospitalization costs.

## 1 Introduction

With an aging population, the incidence of osteoarthritis, osteoporosis, and fractures remains high, leading to an increasing demand for orthopedic surgery. Therefore, selecting a safer and more effective anesthetic approach is essential to ensure smooth procedures and optimal patient outcomes. Postoperative pulmonary complications (PPC) are a major cause of increased postoperative mortality, prolonged length of stay (LOS), and increased hospitalization costs (1). The incidence of PPC in major surgery ranges from 1 to 23% (2), mainly due to changes in the respiratory system following general anesthesia. Conventional pressure-controlled ventilation (PCV), a major component of general anesthesia, increases ventilation in the ventral portion of the lungs while reducing airflow in the dorsal portion. Additionally, laryngeal mask airway (LMA) intubation with spontaneous breathing (SB) has been shown to prevent the redistribution of ventilation (3). Previous studies suggest that preserving SB helps prevent lung injury caused by excessive tidal volume or alveolar pressure, maintains basic lung function, and prevents atelectasis (4, 5). The diaphragm-dependent lung regions exhibit the greatest movement during the retention of SB, while the lack of physiological diaphragmatic motion during PCV leads to reduced dorsal lung ventilation (6, 7). However, some researchers have proposed that SB during general anesthesia contributes to the development of atelectasis and reduction in functional residual vital capacity (8).

Maintaining SB can be challenging because of the need for large doses of opioid analgesics for LMA intubation. Combined with ultrasound-guided peripheral nerve block (PNB), it can provide complete analgesia, reduce intraoperative body movement, significantly decrease the consumption of opioid analgesics, and minimize or even eliminate the need for muscle relaxants. With the development of ultrasound-guided technology, an increasing number of anesthesiologists now prefer PNB as their primary anesthetic technique or adjunct to general anesthesia. Compared with general anesthesia, PNB has some advantages, including prolonged postoperative analgesia, reduced respiratory and circulatory depression, and a reduced risk of in-hospital mortality and PPC (9, 10). Owing to the complexity and diversity of nerve innervation in some orthopedic surgeries, the incomplete analgesic effect of PNB alone is problematic. General anesthesia is usually required to relieve patient fear and anxiety. General anesthesia with LMA intubation is simple and convenient, providing good hemodynamic stability during anesthesia induction and recovery (11). Patients with SB can, through an LMA, reduce airway stimulation, improve airway patency, shorten postoperative anesthesia consciousness recovery time and full awake time to achieve better anesthesia effects (12). Previous studies have shown that LMA can be safely and effectively used in elective short surgeries and that SB results in a faster recovery than PCV (13). However, concerns remain regarding the potential of LMA to increase aspiration risk. While SB with LMA may enhance recovery, its combined use with PNB in orthopedic surgery remains underexplored. Therefore, we conducted a retrospective study to explore the application of general anesthesia with SB-LMA intubation combined with PNB in orthopedic surgery, its effect on PPC, and other relevant outcomes, aiming to provide further insights into optimizing orthopedic anesthesia. This is the first study to demonstrate the cost-effectiveness of the SB-LMA-PNB combination in orthopedic surgery.

## 2 Materials and methods

### 2.1 Study design

This retrospective cohort study was approved by the Ethics Committee of the First Affiliated Hospital of Huzhou University (No. 2024KYLL075-01). It was conducted in accordance with the Declaration of Helsinki and registered in the Chinese Clinical Trial Registry (ChiCTR2500097210). Owing to its retrospective nature, confidentiality of patient data was strictly maintained.

### 2.2 Patients

Clinical data were collected from medical records of patients who underwent elective orthopedic surgeries between January 2021 and August 2024. Inclusion criteria were: (1) age ≥ 18 years old, (2) American Society of Anesthesiologists (ASA) grade 1–3, (3) patients undergoing elective orthopedic surgery (including fracture internal fixation, joint replacement, internal fixation removal, or arthroscopy). (4) All patients that have received combined general anesthesia with LMA intubation and PNB. Exclusion criteria comprised: (1) failure to preserve SB (defined as the inability to restore or sustain normal oxygen saturation (SpO_2_), or coughing/movement interfering with surgery), (2) ≥ 2 surgeries during hospitalization, (3) LOS ≥ 31 days, (4) preoperative respiratory failure, pulmonary infection, myocardial infarction, heart failure, hepatic/renal insufficiency, (5) incomplete electronic medical records. Group assignment was based on anesthesiologist preference and patient-specific factors such as anticipated surgical duration, and comorbidities. The SB group was included patients with low aspiration risk and anticipated short surgeries. Pre-existing pulmonary disease (such as chronic obstructive pulmonary disease (COPD), asthma) and neuromuscular disorders (such as myasthenia gravis) were explicitly documented and compared between groups.

### 2.3 Anesthesia protocol

#### 2.3.1 Peripheral nerve block

All patients fasted preoperatively and underwent standard cardiac monitoring upon entering the operating room, including electrocardiogram, heart rate, blood pressure (BP), SpO_2_, and respiratory rate. Peripheral intravenous access was established for fluid administration. After routine skin disinfection and draping, ultrasound-guided localization was performed based on the surgical site. For the upper limb nerve blocks, 15–20 mL of 0.375% ropivacaine was administered, while 30–40 mL of 0.5% ropivacaine was used for the lower limb nerve blocks. Upper limb blocks included interscalene, supraclavicular and axillary approaches. Within 20 min post-injection, a pinprick test was conducted to assess the sensory and motor blockade in the corresponding innervated areas. General anesthesia was induced only after confirming effective nerve blockade.

All nerve blocks were performed under real-time ultrasound guidance to minimize complications. Although diaphragmatic motion was not routinely monitored (as this is not standard practice in our retrospective setting), we assessed the clinical signs of phrenic nerve involvement, including new-onset dyspnea, transient oxygen desaturation (SpO_2_ decrease > 5% from baseline), or abnormal breath sounds on auscultation. Patients exhibiting these signs would have received an ultrasound or chest radiography to rule out pneumothorax or diaphragmatic paralysis.

#### 2.3.2 General anesthesia protocol

##### 2.3.2.1 Induction

###### 2.3.2.1.1 SB group

Induction was performed using intravenous propofol (1.5–2.5 mg/kg). Upon loss of consciousness, an LMA was inserted orally and connected to a ventilator to preserve the SB. Manual ventilation was administered for 1–2 min if hypoxemia (SpO_2_ < 90%) or apnea occurred until SB resumed.

###### 2.3.2.1.2 PCV group

Induction included intravenous fentanyl (4–5 μg/kg), propofol (2.0–2.5 mg/kg), and rocuronium bromide (0.6–1 mg/kg). After loss of consciousness, the LMA was placed, and PCV was initiated with a tidal volume of 6–8 mL/kg, respiratory rate of 12–14 breaths/min, and inspiratory-to-expiratory ratio of 1:2. The ventilator settings were adjusted to maintain end-tidal carbon dioxide within 35–40 mmHg.

##### 2.3.2.2 Maintenance

###### 2.3.2.2.1 SB group

Anesthesia was maintained with inhaled sevoflurane (2.5–3.0%), with concentrations titrated based on end-tidal carbon dioxide levels. Intermittent boluses of fentanyl (10–20 μg) were administered intraoperatively as needed to deepen anesthesia.

###### 2.3.2.2.2 PCV group

Maintenance involved inhaled sevoflurane (2.5–3.0%) with supplemental fentanyl and rocuronium bromide to sustain sedation, analgesia, and neuromuscular blockade.

###### 2.3.2.2.3 Both groups

Some patients in the two groups received an intravenous infusion of propofol (30–80 μg/kg/min) to deepen anesthesia. All patients received a 50% oxygen-air mixture with continuous monitoring of electrocardiogram, mean arterial pressure, SpO_2_, and end-tidal carbon dioxide. Airway pressure and flow waveforms were assessed throughout the procedure.

##### 2.3.2.3 Postoperative management

Reversal was initiated when clinical signs indicated adequate recovery (sustained SB, eye opening, or ability to lift head ≥ 5 s, tidal volume > 5 mL/kg). Neuromuscular blockade was reversed with neostigmine (0.04 mg/kg) and atropine (0.02 mg/kg), followed by extubation. Patients were transferred to the post-anesthesia care unit for routine oxygen therapy until they met discharge criteria (Aldrete score ≥ 9), after which they returned to the ward.

### 2.4 Data

#### 2.4.1 Data collection

Patient data were extracted from the “Do Care” anesthesia system and electronic medical records at the First Affiliated Hospital of Huzhou University. The following data were collected:

Demographics: Sex, age, and body mass index. Perioperative clinical data included medical history (hypertension, diabetes, cardiovascular disease, hepatic disease, pulmonary disease, neuromuscular disorders, and cerebrovascular disease), American Society of Anesthesiologists physical status classification, surgical procedure, PNB techniques, transient oxygen desaturation events, anesthetic drug dosages, intraoperative blood loss, total fluid intake, surgery duration, anesthesia duration, anesthesia recovery time, adverse events during recovery, numerical rating scale score at 2 h postoperatively, incidence of PPC, anesthesia costs, surgical costs, total hospitalization costs, and LOS.

#### 2.4.2 Definitions

- **Anesthesia recovery time**: Duration from extubation after surgery to post-anesthesia care unit discharge.
- **Adverse events during recovery**: Postoperative hypoxemia, hypotension, or hypertension occurring in the post-anesthesia care unit.
- *Hypoxemia:* SpO₂ < 90% lasting ≥ 10 s or PaO₂/FiO₂ < 300 mmHg (14).
- *Hypotension:* BP > 20% below baseline for ≥ 5 min.
- *Hypertension:* BP > 20% above baseline for ≥ 5 min.
- **PPC**: Occurrence of ≥1 of the following during hospitalization: respiratory infection, respiratory failure, pleural effusion, atelectasis, pneumothorax, bronchospasm, or aspiration pneumonia (15). Although routine postoperative chest imaging was not performed, all patients underwent mandatory clinical monitoring for signs and symptoms suggestive of PPC. These included new or worsening cough, sputum production, fever (>38 °C), auscultatory findings (such as rales or diminished breath sounds), and persistent hypoxemia (SpO_2_ < 90% on room air). Chest radiographs or CT scans were obtained selectively when such clinical indicators suggested pulmonary involvement to confirm the diagnosis. CT scans were only obtained if radiographs were inconclusive.
- **Postoperative pain:** Assessed using the numerical rating score (0 = no pain; 10 = worst pain) 2 h postoperatively.

#### 2.4.3 Outcome measures

- **Primary outcome**: Incidence of PPC.
- **Secondary outcomes**: Total fentanyl consumption, anesthesia recovery time, incidence of adverse events during recovery, LOS, numerical rating scale score at 2 h postoperatively, and anesthesia costs.

### 2.5 Statistical analysis

Data analysis was performed using R version 4.2.2 (R Foundation for Statistical Computing, Vienna, Austria). The normality of continuous variables was assessed using the Shapiro–Wilk test. Normally distributed data are presented as mean ± standard deviation, and intergroup differences were analyzed using Student’s *t*-test. Effect sizes were reported as mean differences (MD) with 95% confidence intervals. Non-normally distributed continuous variables are expressed as median (interquartile range, IQR), and the Wilcoxon rank-sum test was used for group comparisons. Effect sizes were quantified by Cliff’s *δ* and 95% confidence intervals via bootstrap (1,000 iterations). Categorical variables are reported as frequencies (percentages), with differences between groups evaluated using Pearson’s chi-square test or Fisher’s exact test, as appropriate. Effect sizes for binary outcomes were reported as risk differences (RD) with 95% confidence intervals calculated using the Newcombe–Wilson method. A two-tailed *p* < 0.05 was considered statistically significant.

## 3 Results

**Figure 1:**
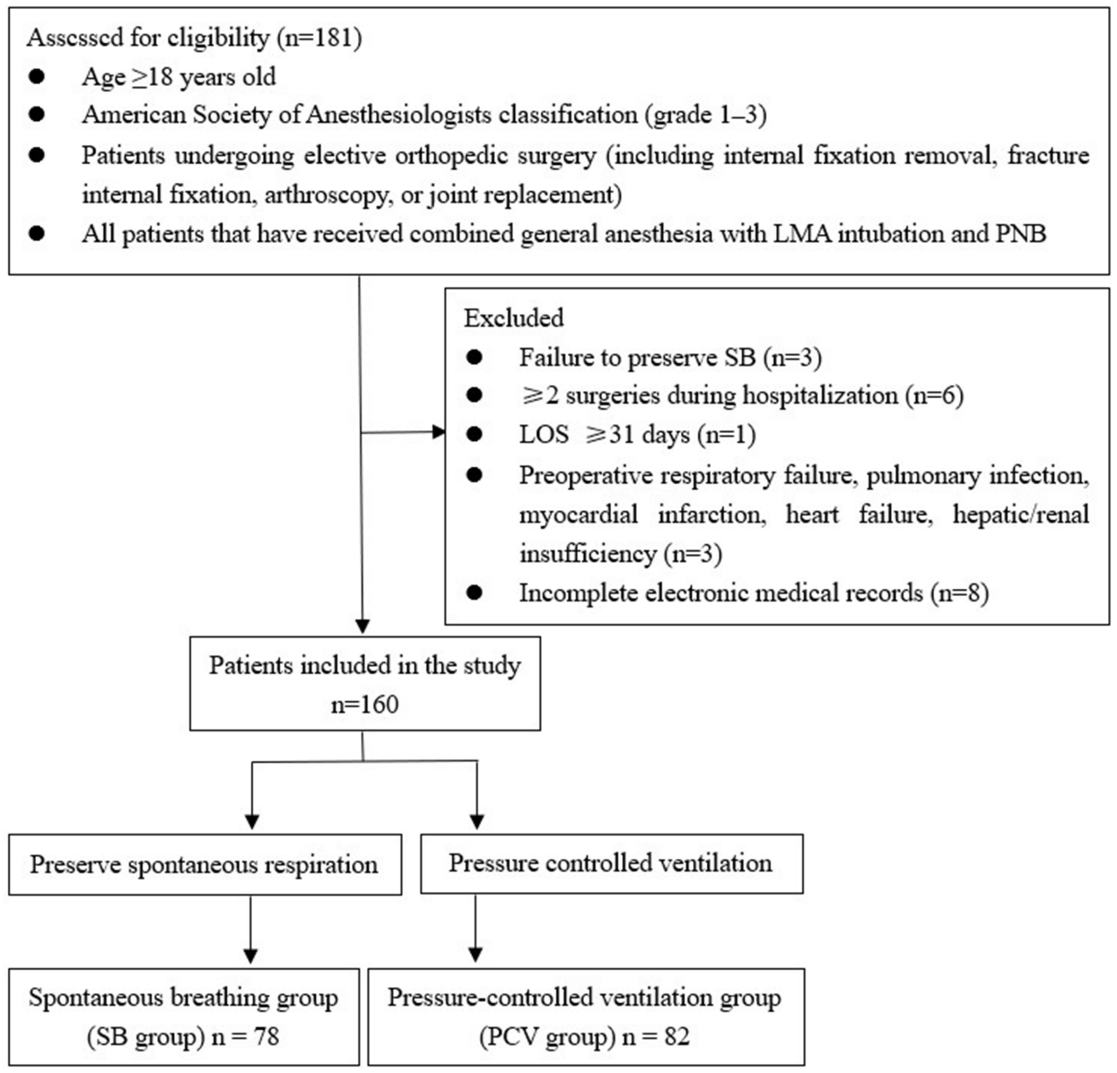
Patients inclusion and exclusion criteria for retrospective analysis.

A total of 181 patients were enrolled in this study. After rigorous screening based on the inclusion and exclusion criteria, 160 patients were included in the final analysis. According to the anesthesia protocol, the enrolled patients were divided into SB and PCV groups for retrospective evaluation (Figure 1). This study focused on comparing the effects of SB and PCV under general anesthesia combined with LMA intubation and PNB during orthopedic surgeries. All enrolled patients underwent elective procedures, including fracture internal fixation (*n* = 66), joint replacement (*n* = 36), internal fixation removal (*n* = 20), and arthroscopy (*n* = 38). All surgeries were completed successfully without conversion to endotracheal intubation.

Comparative analysis between the SB and PCV groups revealed no statistically significant differences (all *p* > 0.05) in baseline demographics, including age, sex, body mass index, medical history (hypertension, diabetes, cardiovascular disease, hepatic disease, pulmonary disease, neuromuscular disorders, and cerebrovascular disease), American Society of Anesthesiologists classification, surgical procedure, intraoperative blood loss, total fluid intake, surgery duration, and anesthesia duration (Table 1). The three pulmonary disease cases in the SB group included two patients with COPD and one with asthma, all with well-controlled symptoms preoperatively.

**Table 1:** Baseline characteristics.

Variable	PCV group (*n* = 82)	SB group (*n* = 78)	*P-*value
Age (yr)	64 (48, 73)	61 (53, 69)	0.468
Sex			0.962
Male	35 (42.7%)	33 (42.3%)	
Female	47 (57.3%)	45 (57.7%)	
Body mass index (kg /m^2^)	24.9 ± 4.2	23.6 ± 3.9	0.057
Hypertension	29 (35.4%)	32 (41.0%)	0.461
Diabetes	11 (13.4%)	6 (7.7%)	0.240
Cardiovascular disease	5 (6.1%)	5 (6.4%)	>0.999
Hepatic disease	0 (0.0%)	2 (2.6%)	0.236
Pulmonary disease	0 (0.0%)	3 (3.8%)	0.114
Neuromuscular disorders	0 (0.0%)	0 (0.0%)	>0.999
Cerebrovascular disease	1 (1.2%)	4 (5.1%)	0.202
ASA grade			0.286
1	37 (45.1%)	27 (34.6%)	
2	44 (53.7%)	48 (61.5%)	
3	1 (1.2%)	3 (3.8%)	
Surgical procedure			0.990
Fracture internal fixation	33 (40.2%)	33 (42.3%)	
Joint replacement	19 (23.2%)	17 (21.8%)	
Internal fixation removal	10 (12.2%)	10 (12.8%)	
Arthroscopy	20 (24.4%)	18 (23.1%)	

PCV, Pressure-controlled ventilation; SB, Spontaneous breathing; ASA, American Society of Anesthesiologists.

Values are *n* (%), mean ± SD or median (interquartile range). The bold values indicate *p* < 0.05.

**Figure 2:**
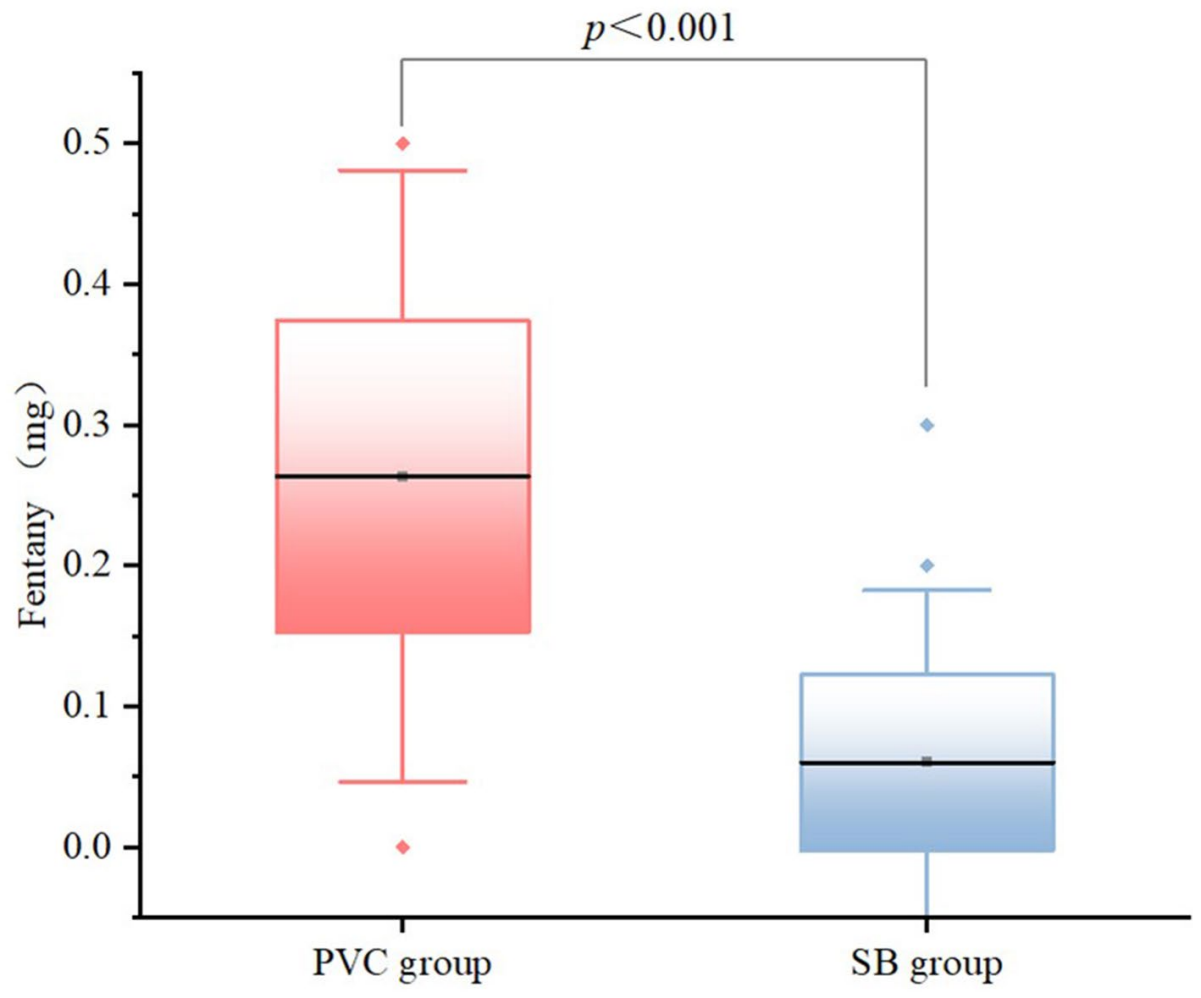
Box plots of fentanyl doses in the PCV group and the SB group. The line inside the box represents the median, box edges represent 25th and 75th percentile and the whiskers represent the minimum and maximum values.

**Figure 3:**
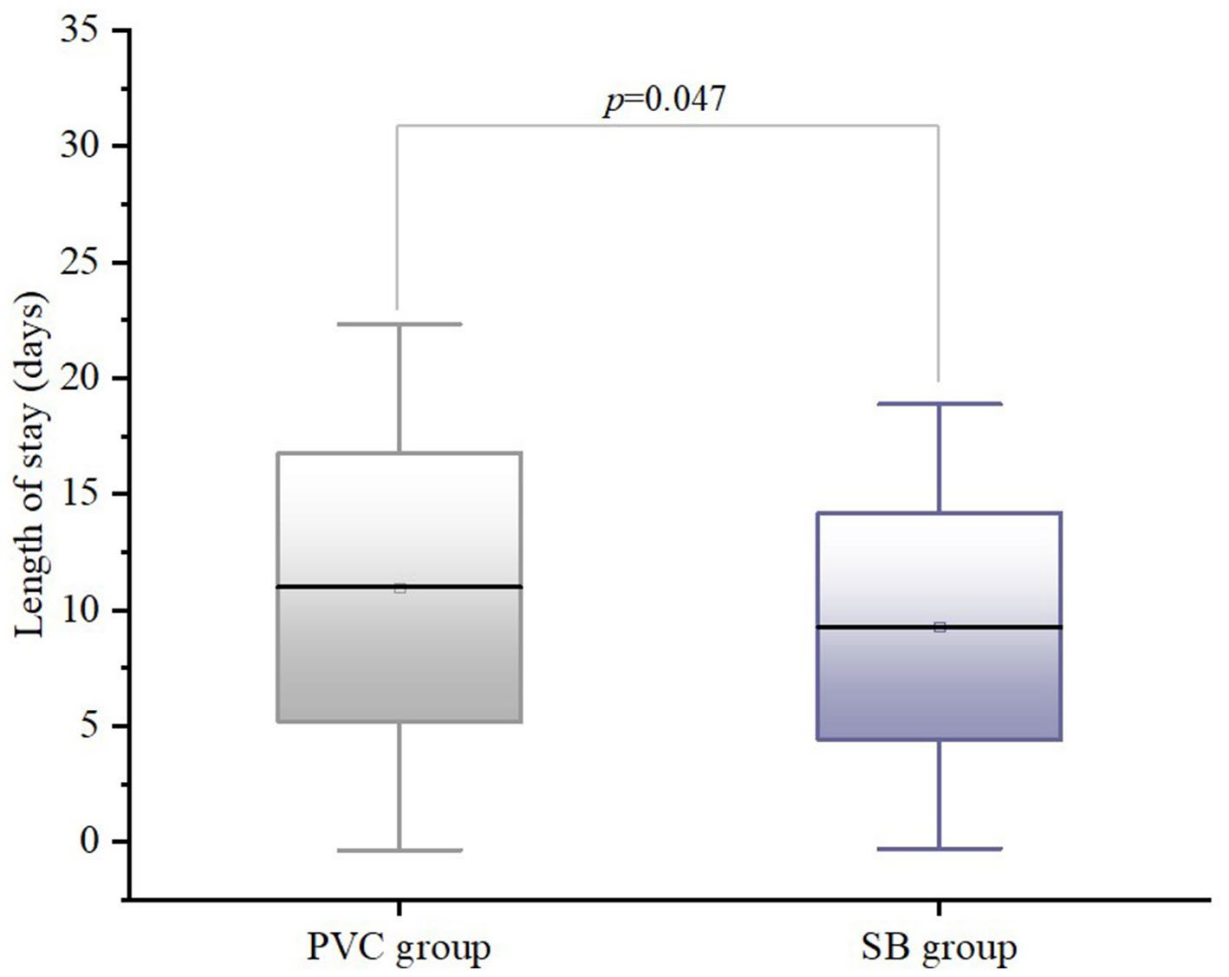
Box plots of length of stay (LOS) in the PCV group and the SB group. The line inside the box represents the median, box edges represent 25th and 75th percentile and the whiskers represent the minimum and maximum values.

Regarding anesthetic agent use, there were significant differences between the groups in fentanyl consumption (*p* < 0.001), with the SB group requiring lower doses (Figure 2). Similarly, propofol administration differed significantly between the groups (*p* < 0.001), with the SB group demonstrating higher dose requirements than the PCV group. Conversely, no significant differences were noted for ropivacaine or sevoflurane use (all *p* > 0.05). Notably, LOS was significantly shorter in the SB group (*p* = 0.047) (Figure 3).

All PNBs were successfully performed under ultrasound guidance. Regarding the distribution of regional anesthesia techniques, Table 2 details the types of PNB performed. Among the 45 patients who underwent upper limb surgeries, the distribution of regional anesthesia techniques was as follows: interscalene approach (*n* = 30, 66.7%), supraclavicular approach (*n* = 13, 28.9%), and axillary approach (*n* = 2, 4.4%). There were no significant differences in block selection between the SB and PCV groups (Table 2). Importantly, no clinically significant complications (such as pneumothorax or symptomatic diaphragmatic paralysis) occurred in any patient who received interscalene or supraclavicular blocks. A few patients (*n* = 5, 3.1%) exhibited transient oxygen desaturation (SpO_2_ 88–92%) during block assessment, which resolved immediately with supplemental oxygen (2–4 L/min via nasal cannula) without requiring further intervention. Postoperative chest radiography was not routinely performed, but no clinical signs of phrenic nerve palsy were documented in medical records during hospitalization.

**Table 2:** Distribution of peripheral nerve block (PNB) techniques and transient oxygen desaturation events.

Variable	PCV group (*n* = 82)	SB group (*n* = 78)	*P-*value
Peripheral nerve block techniques			0.756
Interscalene	18 (22.0%)	12 (15.4%)	
Supraclavicular	7 (8.5%)	6 (7.7%)	
Axillary Approach	1 (1.2%)	1 (1.3%)	
Lower Limb Blocks	56 (68.3%)	59 (75.6%)	
Transient oxygen desaturation	3 (3.7%)	2 (2.6%)	>0.999

PCV, Pressure-controlled ventilation; SB, Spontaneous breathing.

Values are *n* (%), mean ± SD or median (interquartile range). The bold values indicate *p* < 0.05.

Although there were no statistically significant differences in anesthesia recovery time (*p* = 0.178) or PPC incidence (*p* = 0.059), the SB group exhibited numerically shorter recovery intervals of 40 min (IQR: 38, 50) compared to 45 min (IQR: 40, 50) in the PCV group, and reduced PPC rates (0.0% compared to 6.1% in the PCV group). Adverse events during recovery, including postoperative hypoxemia and hypotension, were similar between the groups (all *p* > 0.05). However, the incidence of postoperative hypertension was significantly lower in the SB group (*p* < 0.001). Numerical rating scale score at 2 h postoperatively showed no significant differences between groups (*p* > 0.05). Cost analysis revealed significantly lower total hospitalization costs (*p* = 0.001) and anesthesia costs (*p* < 0.001) in the SB group, whereas surgical costs remained comparable between groups (*p* > 0.05) (Table 3). One patient in the PCV group required transfer to the intensive-care unit because of persistent hypotension during recovery, necessitating vasoactive drug infusion. The patient returned to the general ward the following day. The significant differences in anesthetic drug dosages, incidence of postoperative hypertension, LOS, and hospitalization costs highlight the potential advantages of the SB strategy in optimizing perioperative outcomes for elective orthopedic surgeries.

**Table 3:** Perioperative data.

Variable	PCV group (*n* = 82)	SB group (*n* = 78)	Statistic (95%CI)	*P-*value
Ropivacaine (mg)	75 (75, 100)	75 (75, 100)	δ = 0.014 (−0.15, 0.18)	0.858
Fentanyl (mg)	0.3 (0.2, 0.3)	0.1 (0.0, 0.1)	δ = 0.875 (0.80, 0.92)	**<0.001**
Propofol (mg)	100 (100, 138)	165 (150, 180)	δ = −0.638 (−0.75, −0.50)	**<0.001**
Rocuronium bromide (mg)	40 (5, 40)	0 (0, 0)	δ = 0.561 (0.43, 0.68)	**<0.001**
Sevoflurane (ml)	18 (10, 24)	15 (10, 20)	δ = 0.123 (−0.03, 0.28)	0.171
Blood loss (ml)	20 (10, 50)	18 (5, 50)	δ = 0.129 (−0.03, 0.28)	0.155
Total fluid intake (ml)	1,000 (500, 1,000)	1,000 (500, 1,000)	δ = 0.140 (−0.01, 0.29)	0.092
Surgery duration (min)	85 (50, 110)	67 (40, 101)	δ = 0.167 (0.01, 0.32)	0.069
Anesthesia duration (min)	118 (90, 145)	110 (80, 144)	δ = 0.097 (−0.06, 0.25)	0.290
Anesthesia recovery time (min)	45 (40, 50)	40 (38, 50)	δ = 0.122 (−0.03, 0.28)	0.178
Adverse events during recovery				
Hyoxemia	5 (6.1%)	0 (0.0%)	RD = 0.061 (0.009, 0.113)	0.059
Hypotension	7 (8.5%)	13 (16.7%)	RD = −0.082 (−0.184, 0.020)	0.120
Hypertension	27 (32.9%)	5 (6.4%)	RD = 0.265 (0.150, 0.380)	**<0.001**
Numerical rating scale score at 2 h postoperatively	2.0 (0.0, 3.0)	1.0 (0.0, 3.0)	δ = 0.032 (−0.12, 0.19)	0.709
Incidence of PPC	5 (6.1%)	0 (0.0%)	RD = 0.061 (0.009, 0.113)	0.059
Total hospitalization cost (CNY)	27804.5 ± 12539.0	21974.2 ± 9909.0	MD = 5830.4 (2289.8, 9370.9)	**0.001**
Anesthesia costs	1730.4 ± 187.1	1563.4 ± 349.0	MD = 167.0 (80.2, 253.8)	**<0.001**
Surgical costs	3984.3 ± 1750.8	3637.2 ± 1526.7	MD = 347.1 (−166.9, 861.1)	0.183
LOS (d)	11.0 ± 5.8	9.3 ± 4.9	MD = 1.7 (0.2, 3.3)	**0.047**

PCV, Pressure-controlled ventilation; SB, Spontaneous breathing; CI, Confidence Intervals; PPC, postoperative pulmonary complications; LOS, Length of stay.

Values are *n* (%), mean ± SD or median (interquartile range). Effect sizes for non-normally distributed continuous variables were quantified by Cliff’s δ with 95% CI via bootstrap (1,000 iterations). Cliff’s δ interpretation: |δ| < 0.147 (negligible), 0.147 ≤ |δ| < 0.33 (small), 0.33 ≤ |δ| < 0.474 (medium), |δ| ≥ 0.474 (large). For normally distributed variables, mean differences (MD) with 95% CI are reported. For binary outcomes, risk differences (RD) with 95% CI calculated by Newcombe-Wilson method are reported. The bold values indicate *p* < 0.05.

## 4 Discussion

The incidence of PPC during major surgeries ranges from 1 to 23% (2). Following major surgeries under general anesthesia, respiratory function can take up to 6 weeks to return to preoperative levels (2, 16). Patients who develop PPC have a significantly higher 90-day mortality rate (24.4%) compared to those without PPC (1.2%) (2). PCV during general anesthesia plays a significant role in the development of PPC. LMA is a widely used airway management method that reduces airway irritation, improves patency. However, concerns remain regarding its potential to increase aspiration risk. A retrospective analysis of 65,712 patients demonstrated that LMA combined with intraoperative PCV did not increase aspiration rates compared to endotracheal intubation (17). Additionally, lung ultrasound studies have revealed that LMA use is associated with fewer and milder PPC, particularly atelectasis compared to endotracheal intubation (18). In our study, LMA use was strictly avoided in high-aspiration-risk patients, and the overall incidence of PPC following elective orthopedic surgery was 3.1%, which is consistent with previous reports. The balanced distribution of interscalene/supraclavicular blocks between groups mitigates concerns about confounding effects from block-specific risks. The precise distribution of ultrasound-guided PNBs in our cohort likely contributed to reduced opioid requirements in the SB group. This targeted approach ensures adequate analgesia while minimizing systemic drug exposure, aligning with enhanced recovery principles (10). In addition, by avoiding the use of neuromuscular blocking agents (NMBAs), the SB strategy significantly reduces healthcare costs for patients.

In his comprehensive review of LMA applications, Hunter proposed that the intraoperative use of NMBAs, regardless of whether LMA or endotracheal intubation is used, increases the risk of PPC. This association may stem from the necessity of PCV when NMBAs are administered, as mechanical ventilation can impair diaphragmatic contractility (19). Reber et al. further provided critical insights through diaphragmatic motion analysis and demonstrated cephalad displacement of the diaphragm during mechanical ventilation (20). A previous animal experimental study has demonstrated that preserving SB during mechanical ventilation not only improves ventilatory function but also attenuates selected biomarkers of ventilator-induced lung injury (21). Electrical impedance tomography studies in elective orthopedic surgeries under LMA-general anesthesia have revealed that PCV induces ventral redistribution of pulmonary ventilation, whereas SB effectively prevents ventilation heterogeneity (3). Our study protocol uniquely combined SB preservation with complete avoidance of NMBAs. Although statistical significance was not reached for PPC incidence between groups (*p* = 0.059), the SB group demonstrated a clinically relevant reduction in PPC incidence compared to the PCV group (0.0% compared to 6.1% in the PCV group). This discrepancy could be primarily attributed to the requirement for radiological confirmation of pneumonia, which is not routinely performed postoperatively, potentially under-diagnosing mild cases. Additionally, preoperative antibiotic prophylaxis in some orthopedic surgeries may have influenced the outcomes. Our PPC diagnostic protocol mirrors pragmatic clinical workflows. Although surveillance radiography may increase detection rates, its clinical utility remains debated. We hypothesize that preserving the intraoperative SB helps maintain natural physiological breathing mechanics, ensuring adequate oxygenation and ventilation while reducing iatrogenic perturbations from non-physiological ventilation strategies. Our study also revealed a statistically significant intergroup difference in the incidence of postoperative hypertension during recovery (*p* < 0.001), with the SB group demonstrating significantly lower rates than the PCV group. This suggests that preserving SB may stabilize hemodynamics during recovery more effectively than PCV. This phenomenon may be attributed to the physiological respiratory mechanics of SB, where intrathoracic pressure fluctuations associated with natural breathing facilitate venous return, potentially reducing adverse circulatory effects.

In recent years, the application of LMA with preserved SB has gained attention in thoracic anesthesia. Researchers have demonstrated that SB-LMA anesthesia combined with thoracic paravertebral blockade for thoracoscopic bulla resection is safe and feasible. This approach avoids the risks associated with double-lumen endotracheal intubation while providing effective postoperative analgesia (22). Furthermore, a study evaluating LMA-general anesthesia with preserved SB and thoracic paravertebral blockade in video-assisted thoracic surgery observed that it reduced hospitalization duration, lowered postoperative visual analog scale scores, had fewer complications, and accelerated recovery (23). Compared to conventional anesthesia, LMA serves as a viable alternative to endotracheal intubation, ensuring preserved SB, simplifying airway management, and minimizing complications related to intubation trauma and residual neuromuscular blockade. In our study, the combination of SB-LMA intubation and PNB in elective orthopedic surgeries for adults significantly reduced LOS (*p* = 0.047), anesthesia costs (*p* < 0.001), and total hospitalization costs (*p* = 0.001). However, given that LOS is influenced by multiple factors, prospective studies with multicenter designs are needed to further clarify these findings. Notably, this strategy significantly reduced opioid consumption without increasing the incidence of postoperative analgesic insufficiency, primarily because of the adjunctive benefits of PNB. These outcomes align with the enhanced recovery after surgery principles, which emphasize reduced opioid use, shortened hospitalization, effective postoperative analgesia, and reduced medical costs.

The residual neuromuscular blockade remains a well-recognized concern (24). According to *Miller’s Anesthesia*, RNMB-related PPC continues to significantly contribute to morbidity and mortality. Sugammadex, a synthetic γ-cyclodextrin derivative, acts as a selective antagonist for non-depolarizing steroid-based NMBAs, rapidly restoring SB and motor function to enhance anesthetic safety. However, its use presents challenges, such as the need to reestablish neuromuscular blockade within 24 h after reversal and imprecise dosing without quantitative neuromuscular monitoring [such as train-of-four (TOF)], particularly in patients with obesity (25). Despite reports of adverse effects such as hypersensitivity reactions, the impact of sugammadex on healthcare costs remains unclear (26–29). Notably, most orthopedic surgeries do not require deep neuromuscular blockade. In our study, preserving SB eliminated the need for NMBAs, thereby avoiding residual neuromuscular blockade-related risks. Furthermore, sugammadex was not available at our institution during the data collection period. Regarding neuromuscular blockade reversal in the PCV group, the use of neostigmine/atropine without routine TOF monitoring warrants discussion. Although quantitative monitoring is ideal, our protocol employed rigorous clinical assessments including respiratory rate and pattern monitoring, tidal volume measurement and a sustained head lift test. Importantly, no clinical manifestations of residual blockade were observed during recovery.

A prospective randomized controlled study demonstrated that SB ventilation can be safely employed for brief surgical procedures in high-turnover operating environments, whereas PCV may prolong anesthesia recovery time (30). Another investigation revealed that LMA general anesthesia with preserved SB combined with PNB for intertrochanteric fracture surgery in older patients significantly shortened anesthesia recovery time, extended analgesic duration, and minimized hemodynamic disturbances (12). Although our study did not identify statistically significant differences in anesthesia recovery time between the groups, the SB group exhibited numerically shorter recovery intervals of 40 min (IQR: 38, 50) compared to the PCV group of 45 min (IQR: 40, 50). This marginal difference might be partially attributed to the absence of quantitative TOF monitoring during neuromuscular blockade reversal in the PCV group. Without TOF guidance, clinicians may adopt more conservative approaches to ensure safe recovery, potentially delaying extubation until unequivocal clinical recovery signs are observed.

This study has some limitations. First, as a single-center retrospective study, it carries inherent risks of potential bias in data collection and case selection. Additionally, the limited sample size may have compromised the statistical power and generalizability of the findings. Third, PPCs were diagnosed primarily based on clinical criteria rather than protocolized imaging. Although this reflects real-world practice and prioritizes clinically relevant events, it may underestimate radiologic-only abnormalities. Nevertheless, such subclinical findings rarely impact recovery pathways (2). Finally, diaphragmatic function was not quantitatively assessed due to the retrospective design. We screened for clinical indicators of phrenic nerve blockade (dyspnea, desaturation, auscultation); however, subtle diaphragmatic weakness could have been missed. Nevertheless, the absence of clinically significant events suggests that any undetected effects were unlikely to impact outcomes. Future large-scale, multicenter prospective studies with preoperative randomization are warranted to eliminate selection bias and validate these findings, ultimately optimizing anesthetic strategies. Further, when implementing combined LMA general anesthesia with preserved SB and PNB, we emphasize the necessity for continuous monitoring of the ventilatory status throughout the procedure. Special attention should be directed toward preventing LMA displacement and respiratory depression to maintain adequate oxygenation.

Although our single-center design limits broad extrapolation, the cohort included diverse orthopedic procedures and ASA grade 1–3 patients, supporting applicability to similar settings. In conclusion, general anesthesia with SB-LMA intubation combined with PNB is a safe and feasible approach for elective orthopedic surgeries in adults. This strategy significantly shortens the LOS (*p* = 0.047) and reduces hospitalization costs (*p* = 0.001), demonstrating its potential to optimize perioperative outcomes while alleviating economic burden. This approach may be advantageous in resource-limited settings and for high-risk patient populations, including the elderly and those with pulmonary comorbidities, but it necessitates vigilant airway monitoring.

## Data Availability

The raw data supporting the conclusions of this article will be made available by the authors, without undue reservation.
